# Outcomes of Concurrent Endocarditis and Periprosthetic Joint Infection: A Retrospective Case Series of 16 Patients

**DOI:** 10.7759/cureus.24139

**Published:** 2022-04-14

**Authors:** Tyler J Humphrey, Daniel Marchwiany, Mehdi S Salimy, Sandra B Nelson, Hany S Bedair, Christopher M Melnic

**Affiliations:** 1 Orthopaedic Surgery, Massachusetts General Hospital, Boston, USA; 2 Infectious Disease, Massachusetts General Hospital, Boston, USA

**Keywords:** two-stage revision and reconstruction, explant, morbidity, mortality, outcomes, total joint arthroplasty, periprosthetic joint infection, endocarditis

## Abstract

Introduction

Concurrent diagnosis of periprosthetic joint infection (PJI) of total hip arthroplasty (THA) or total knee arthroplasty (TKA) with infectious endocarditis is a devastating clinical scenario infrequently documented in the literature. To date, no studies have fully described the orthopedic and infectious outcomes of patients with these concurrent diagnoses. The purpose of this study was to conduct a case series of patients with these diagnoses and document the orthopedic and infectious outcomes so that surgeons may effectively counsel patients regarding the gravity of the condition and the expected course of treatment.

Methods

This study is a retrospective case series using patient data from five hospitals within an academic healthcare system in the northeastern United States. Cases of concurrent endocarditis and THA or TKA PJI with a minimum of one-year follow-up were identified from January 2000 to January 2021. Basic statistics such as means, standard deviations, and percentages were used to identify trends within our series. Kaplan-Meier survivorship curves with log-rank tests were performed to determine if there were any differences in two-year mortality and joint survival (defined as needing explant) between patients who had cardiac surgery prior to surgical management for their PJI and those who had surgical management for PJI prior to cardiac surgery.

Results

A total of 18 joints in 16 patients with endocarditis and concurrent TKA or THA PJI were identified. All PJIs were managed surgically, with 14/18 (77.77%) of joint infections initially being managed by debridement, antibiotics, and implant retention (DAIR) and 4/18 (22.22%) of joint infections initially being managed by explant. Within the first six months of PJI diagnosis, 25% (4/16) of patients died of complications related to their infection, and one additional patient died of bacteremia just over a year after the initial PJI diagnosis. Of the 18 PJIs, 72.23% (13/18) had treatment failure, defined as any outcome equal to or worse than requiring chronic suppressive antibiotics for the infection. Due to low statistical power, we were not able to identify any differences in two-year mortality from PJI diagnosis (p=0.311) or joint survival (in terms of requiring explant) (p=0.420) depending on whether cardiac surgery or DAIR was performed first.

Conclusions

Concurrent infectious endocarditis and prosthetic joint infection is associated with high morbidity and mortality. Patients with these concurrent infections should be counseled that not only the associated mortality rate is high, but also the surgical treatment of their PJI has a high rate of treatment failure, including an explant following an initial DAIR, an explant with retained spacer, or a requirement of lifelong antibiotic suppression.

## Introduction

Periprosthetic joint infection (PJI) of total hip arthroplasty (THA) or total knee arthroplasty (TKA) is a potentially devastating complication that occurs in approximately 1%-2% of patients undergoing the procedures [1]. The infectious source of PJI is commonly acquired at the time of surgery or as a result of hematogenous seeding from a distant source. Hematogenous infection has been noted to occur in anywhere from 20% to 30% of PJI cases [2,3]. Nevertheless, the exact source of hematogenous infection is not always identified, with some studies suggesting identification rates of around 68% [4]. One of the possible sources of hematogenous PJI, especially in the setting of multiple simultaneous PJI, is seeding from infectious endocarditis [5,6]. Concurrent PJI and infectious endocarditis represent a rare but significant clinical scenario, as the one-year mortality for total joint arthroplasty (TJA) PJI may approach 3%-8% and the six-month mortality for infectious endocarditis may be as high as 27% [7-9]. Furthermore, patients with these concurrent infections may require both surgery to manage their PJI (debridement, antibiotics, and implant retention (DAIR) or explant of the prosthesis) and cardiac surgery for a variety of indications, such as in the setting of acute congestive heart failure (CHF), embolic phenomena, hemodynamic instability, or prosthetic valve endocarditis especially due to *Staphylococcus aureus* [10,11].

Although prior studies have described that endocarditis may ultimately be found as the source of infection in TJA PJI, most reporting has been in the setting of case reports or as a single variable in retrospective cohort studies with less than 15 cases of endocarditis included [4,5,12]. Furthermore, a thorough characterization of the orthopedic and infectious outcomes of these patients has not been described. Thus, the goal of our study was to characterize the clinical presentation of patients with concurrent PJI and infectious endocarditis and describe the orthopedic and infectious outcomes of these patients in a case series of 16 patients. As patients with these contemporaneous diagnoses are likely to suffer high morbidity and mortality, an understanding of the trends of their clinical courses is needed to sufficiently counsel patients and their families.

## Materials and methods

This is an IRB-approved, retrospective case series using data from the electronic medical records of patients from five hospitals within an academic healthcare system in the northeastern United States. Our institutional database was queried to identify all patients over the age of 18 who had a primary or revision TKA or THA in place (using Current Procedural Terminology (CPT) codes 27447, 27487, 27486, 27130, 27137, 27134, and 27138) and who were then subsequently diagnosed with any of the three endocarditis subtypes native valve endocarditis, prosthetic valve endocarditis, or endocarditis due to a cardiac device (e.g., pacemaker, implantable cardioverter-defibrillator (ICD), and cardiac resynchronization therapy device) (International Classification of Diseases (ICD)-9 and ICD-10 codes I33, I38, I39, T82.6, T82.7, 421, 424, and 996.0) and were also diagnosed with PJI of the hip or knee (ICD-9 and ICD-10 codes 996.6, T84.5, T84.6, T84.7, T84.8, and T84.9). For our study period of January 2000 to January 2021, this query resulted in 330 possible patients. All patients were chart reviewed to confirm that the correct temporal sequence of TJA, endocarditis, and PJI (concurrent with the endocarditis episode) was present.

All patients were also confirmed to have a minimum of one-year clinical follow-up. We also chart reviewed patients to confirm that the three subtypes of endocarditis were diagnosed according to the modified Duke criteria [13] and to confirm that the diagnosis of PJI was made according to the most updated version of the Musculoskeletal Infection Society (MSIS) criteria [14,15]. For patient cases to meet our study’s inclusion criteria, we confirmed that the microbiologic organism causing the endocarditis (by blood culture or tissue/device culture) and the PJI (by aspirate or intraoperative tissue culture) were the same organism, which would suggest that the distant infections were truly linked. Patients were excluded from the final cohort in our case series if patients did not meet the inclusion criteria or had insufficient chart information to confirm all of the required diagnoses and temporal constraints. Following the chart review, 18 TJAs in 16 patients were identified.

For each of the patients identified for our final cohort, a variety of demographic, surgical, infectious, and orthopedic variables were obtained. For each patient, data on age, sex, body mass index (BMI), joint (hip or knee), classification of TJA (primary or revision), and all comorbidities listed in the chart, including a history of septic arthritis, diabetes mellitus, hypertension, immunosuppressive medication prescription (systemic chemotherapy, immunosuppressants, or daily steroids), cancer diagnoses, cerebrovascular accident/stroke diagnoses, congestive heart failure, major depressive disorder, rheumatoid arthritis, smoking history, and alcohol use history, were obtained. For each patient, we also obtained data related to the hospital presentation of their concurrent PJI and endocarditis admission; thus, we documented the following: the number of joints infected, erythrocyte sedimentation rate (ESR) at presentation, C-reactive protein (CRP) at presentation, whether symptoms were acute (<4 weeks), if there were documented recent invasive procedures (such as dental procedures), PJI temporal classification related to the most recent TJA (early (<3 months), delayed (3-24 months), or late (>24 months)) [16], whether there was new-onset joint pain after an uneventful recovery from the index TJA, if patients were septic on initial presentation, if there were any signs of local inflammation (warmth, erythema, and edema) at the affected joint, fever of >38°C, and a description of the intraoperative findings by the orthopedic surgeon managing the PJI.

Regarding infectious and orthopedic outcomes, we documented the occurrence and dates of the diagnosis of endocarditis, diagnosis of PJI, and surgery for the management of PJI, including the following: DAIR, prosthesis explant with antibiotic spacer, full two-stage revision with reimplantation of prosthesis, resection arthroplasty (Girdlestone arthroplasty), cardiac surgery, and repeat cardiac surgery (if applicable). We documented the infectious organism of the endocarditis/PJI and whether the organism was a multidrug-resistant organism (MDRO), which we defined as organisms with resistance to three or more antibiotic classes during susceptibility testing or the presence of methicillin-resistant *Staphylococcus aureus* (MRSA) [17]. We also noted the antibiotics prescribed after the hospitalization for the infections in terms of antibiotic type, duration, and route of administration and whether antibiotics were prescribed for the suppression of infection for >6 months duration. We lastly documented the ultimate outcome of the infections for each patient, as well as the dates of death during the study period. Treatment failure for PJI management was defined as a tier 2 to tier 4 outcome using outcome tiers described by Fillingham et al. [18]. The outcome tiers we utilized for our study can be viewed in Table 1.

**Table 1:** Classification Tiers of Treatment Outcomes for TJA PJI The classification tiers are according to Fillingham et al. [18]. TJA: total joint arthroplasty; PJI: periprosthetic joint infection

PJI outcome tiers
Tier 1: Infection control with no continued antibiotic therapy
Tier 2: Infection control with the patient on suppressive antibiotic therapy
Tier 3: Need for reoperation and/or revision and/or spacer retention (assigned to subgroups A, B, C, D, E, and F based on the type of reoperation)
A: Aseptic revision at >1 year from the initiation of PJI treatment
B: Septic revision (including debridement, antibiotics, and implant retention (DAIR)) at >1 year from the initiation of PJI treatment (excluding amputation, resection arthroplasty, and arthrodesis)
C: Aseptic revision at ≤1 year from the initiation of PJI treatment
D: Septic revision (including DAIR) at ≤1 year from the initiation of PJI treatment (excluding amputation, resection arthroplasty, and arthrodesis)
E: Amputation, resection arthroplasty, or arthrodesis
F: Retained spacer
Tier 4: Death (assigned to subgroups A or B)
A: Death ≤1 year from the initiation of PJI treatment
B: Death >1 year from the initiation of PJI treatment

Statistical analysis

Simple descriptive statistics, such as means, averages, standard deviations, and percentages, were calculated for variables that we obtained in order to identify trends within our cohort. In addition, Kaplan-Meier survivorship curves with log-rank tests were performed to determine if there were any differences in two-year mortality and joint survival (defined as needing explant) between patients who had cardiac surgery prior to surgical management for their PJI and patients who had surgical management for their PJI prior to cardiac surgery. Only patients who had cardiac surgery were included in this Kaplan-Meier survivorship curve analysis (n=13). The p-value for significance was set as p<0.05 for our study. Statistical analyses were performed using the SPSS software for Windows version 26 (IBM Corporation, Armonk, NY, USA).

## Results

Patient demographics

With our inclusion and exclusion criteria, we identified 16 patients with a history of primary or revision THA or TKA who developed concurrent native valve, prosthetic valve, or cardiac device endocarditis and PJI. Table 2 displays the general demographics of our case series. The average age of patients in our cohort at the time of PJI diagnosis was 69.30±9.36 years, with 10 male and six female patients represented. The average BMI in our cohort was 31.65±7.05 kg/m^2^, 56.25% of patients were smokers, 37.5% of patients were diabetic, 62.5% had peripheral artery disease, and 6.25% of patients had a history of septic arthritis. Interestingly, no patients were found to have ever abused injection drugs. Lastly, the average follow-up duration was 43.36 (range: 0.36-143.2) months in our cohort, which was inclusive of patients who died prior to the minimum 12-month follow-up for our study.

**Table 2:** Patient Demographics PJI: periprosthetic joint infection; n/a: not applicable; PAD: peripheral artery disease; IDU: injection drug abuse; CHF: congestive heart failure

Patient number	History of PJI or septic arthritis prior to new PJI	Comorbidities (all)	Diabetes mellitus	Hypertension	Immunosuppressive medications	Notable medications	Cancer	Cerebrovascular accident/stroke	PAD	Smoker	IDU	CHF	Major depressive disorder	Alcohol use	Rheumatoid arthritis
1	No	Atrioventricular block with pacemaker placement, rheumatoid arthritis, tracheomalacia with tracheostomy	No	Yes	Yes	Methotrexate, leucovorin	No	No	Yes	Yes	No	No	No	No	Yes
2	No	Multilevel lumbar fusion, chronic low back pain, diabetes mellitus, hypertension, hyperlipidemia, coronary artery disease	Yes	Yes	No	n/a	No	No	Yes	No	No	No	No	No	No
3	No	Congestive heart failure, coronary artery disease, hypertension, atrial flutter with permanent pacemaker placement, diabetes mellitus	Yes	Yes	No	n/a	No	No	Yes	Yes	No	Yes	No	No	No
4	No	Chronic lymphocytic leukemia, hypertension, peripheral artery disease, congestive heart failure, coronary artery disease, atrial fibrillation, deep venous thrombosis, diabetes mellitus, hyperlipidemia, gastroesophageal reflux disease	Yes	Yes	No	n/a	Yes	Yes	Yes	No	No	Yes	No	No	No
5	No	Diabetes mellitus, obstructive sleep apnea, atrial fibrillation, hypertension, hyperlipidemia, coronary artery disease, aortic stenosis, ascending aortic aneurysm status post-aortic valve replacement and aortic plication	Yes	Yes	No	Apixaban, amiodarone	No	No	Yes	Yes	No	Yes	No	Yes	No
6	No	Human immunodeficiency virus with low CD4 count, end-stage renal disease, hemophilia, factor IX deficiency, hepatitis C, cirrhosis, diabetes mellitus, spontaneous bacterial peritonitis	No	No	No	Acyclovir, ciprofloxacin, supplemental factor IX	No	No	No	No	No	No	No	No	No
7	No	Cardiomyopathy with implantable cardioverter-defibrillator, diabetes mellitus	Yes	Yes	No	Atorvastatin, carvedilol, divalproex, empagliflozin, insulin, metformin, sacubitril-valsartan	No	Yes	No	Yes	No	Yes	Yes	Yes	No
8	No	Chronic kidney disease, hypertension, hyperlipidemia, coronary artery disease, sick sinus syndrome, atrial fibrillation with pacemaker, ulcerative colitis	No	Yes	No	Balsalazide	No	No	Yes	Yes	No	No	No	No	No
9	Yes, of same prosthetic joint but a different organism	Breast cancer status post-radiation therapy, mitral valve disease status post- bioprosthetic mitral valve, atrial fibrillation	No	No	No	Exemestane	Yes	Yes	No	No	No	No	No	No	No
10	No	Prostate cancer, anemia, atherosclerotic cardiovascular disease, glaucoma, neuropathy	No	Yes	No	Atorvastatin, ezetimibe, furosemide, gabapentin, omeprazole	Yes	No	Yes	Yes	No	No	No	Yes	No
	No	Prostate cancer, anemia, atherosclerotic cardiovascular disease, glaucoma, neuropathy	No	Yes	No	Atorvastatin, ezetimibe, furosemide, gabapentin, omeprazole	Yes	No	Yes	Yes	No	No	No	Yes	No
11	No	Coronary artery disease, atrial fibrillation, complete heart block status post-permanent pacemaker placement, mitral valve repair with porcine valve for severe mitral regurgitation, congestive heart failure, history of diverticular bleed, breast cancer status post-mastectomy	No	No	No	Amiodarone	No	No	Yes	No	No	Yes	No	No	No
12	No	Aortic stenosis status post-bioprosthetic valve replacement, atrial flutter, lumbar discectomy, major depressive disorder, obesity, obstructive sleep apnea, stroke, hypertension	No	Yes	No	n/a	No	Yes	No	Yes	No	No	Yes	No	No
	No	Aortic stenosis status post-bioprosthetic valve replacement, atrial flutter, lumbar discectomy, major depressive disorder, obesity, obstructive sleep apnea, stroke, hypertension	No	Yes	No	n/a	No	Yes	No	Yes	No	No	Yes	No	No
13	No	Coronary artery disease, history of bioprosthetic aortic valve replacement, carotid endarterectomy, congestive heart failure, diabetes mellitus	Yes	Yes	No	Insulin	No	Yes	Yes	Yes	No	No	Yes	No	No
14	No	Mitral valve prolapse	No	No	No	n/a	No	No	No	No	No	No	No	No	No
15	No	Dilated nonischemic cardiomyopathy, atrial fibrillation status post-implantable cardioverter-defibrillator placement, alcohol abuse, hypertension, total hip replacement with chronic PJI on suppressive antibiotics	No	Yes	No	Minocycline for chronic suppressive antibiotics, rivaroxaban, amiodarone, omeprazole, quetiapine	Yes	No	No	Yes	No	Yes	Yes	Yes	No
16	No	Hypertension, hyperlipidemia, coronary artery disease, endocarditis, chronic kidney disease, gout, aortic valve replacement	No	Yes	No	Allopurinol, diltiazem, ezetimibe	No	No	Yes	No	No	No	No	No	No

Characteristics of patient presentations

Table 3 depicts the characteristics of patient presentations in our cohort. In our cohort, there were 14 (87.5%) patients with PJI affecting a single joint and two (12.5%) patients presenting with multiple concurrent PJI. Of the PJIs, there were 12 knees and six hips. One patient was diagnosed with a knee PJI and concurrent native shoulder septic arthritis, although we excluded the native shoulder septic arthritis from our outcomes analysis. This resulted in a total of 18 total hip and total knee arthroplasties that were infected in 16 patients. In terms of inflammatory markers, the average ESR at PJI diagnosis was 64.00±36.35 mm/hour (reference: 0-13 mm/hour), and the average CRP at PJI diagnosis was 124.31±72.81 mg/L (reference: <8 mg/L).

In addition, 62.5% of patients had symptoms of PJI and endocarditis for less than four weeks prior to presentation to the hospital. We also found that 81.25% of patients experienced new-onset joint pain after an uneventful recovery from their index TJA and that 62.5% of patients were septic on presentation. In terms of temporal PJI classification, 87.5% of patients had a PJI temporally defined as “late,” but only 18.75% of patients had a documented invasive procedure as a possible impetus for a hematogenous infection. In addition, we found that all patients in our cohort had signs of local inflammation at the affected joint upon presentation, and 93.75% of patients had a fever documented at presentation greater than 38°C. All patients had surgical management of their PJI, with 9/18 (50%) of joints demonstrating intraoperative purulence.

Regarding the timing of diagnoses, eight (50%) patients had their hip or knee PJI diagnosed prior to endocarditis, while the remaining 50% of patients were diagnosed with endocarditis prior to PJI. In the two patients with multiple simultaneous PJI diagnoses, the diagnoses of PJI were made on the same day of hospital admission.

**Table 3:** Characteristics of Patient Presentations *Negative value means PJI was diagnosed prior to endocarditis. PJI: periprosthetic joint infection; BMI: body mass index (kg/m^2^); ESR: erythrocyte sedimentation rate (reference: 0-13 mm/hour); CRP: C-reactive protein (reference: <8 mg/L)

Patient number	Age at PJI diagnosis	Sex	BMI	Type of prosthetic joint infected	ESR at PJI diagnosis (mm/hour)	CRP at PJI diagnosis (mg/L)	Acute symptoms (<4 weeks)	Documented dental procedure or other invasive procedure prior to endocarditis	PJI temporal classification: early (<3 months), delayed (3-24 months), or late (>24 months)	New-onset joint pain after an uneventful recovery	Sepsis on initial endocarditis presentation	Local signs of knee or hip inflammation (erythema and warmth)	Fever above 38°C	Intraoperative findings of PJI management surgery	Days from infected cardiac/vascular device to PJI*
1	76.39	Female	25	Prosthetic knee	23	52.50	Yes	Pacemaker exchange two weeks prior	Late	Yes	Yes	Yes	Yes	No abnormal findings	15
2	75.09	Female	43	Prosthetic knee	Unknown	Unknown	Yes	No	Late	Yes	Yes	Yes	Yes	Purulence	-5
3	70.52	Male	35	Prosthetic hip	110	24	No	No	Late	Yes	No	Yes	Yes	Purulence, loosening	324
4	82.87	Male	28	Prosthetic hip	73	223	Yes	No	Late	Yes	Yes	Yes	Yes	Purulence	6
5	69.87	Male	41	Prosthetic knee	53	189.60	Yes	No	Late	Yes	No	Yes	Yes	Straw-colored fluid	-5
6	54.45	Male	30	Prosthetic knee	Unknown	Unknown	Yes	No	Late	Yes	Yes	Yes	Yes	Large coagulative hematoma	55
7	57.93	Male	32	Prosthetic knee	28	99	Yes	Foot ulcer debridement three months prior	Late	Yes	Yes	Yes	Yes	Gross purulence	6
8	79.99	Male	25	Prosthetic knee	5	76.40	Yes	No	Late	Yes	No	Yes	Yes	Copious purulent joint fluid	-3
9	59.09	Female	28	Prosthetic knee	Unknown	Unknown	No	No	Delayed	No	Yes	Yes	Yes	No abnormal findings	-143
10	78.89	Male	25.50	Prosthetic knee	104	147	No	No	Delayed	No	Yes	Yes	Yes	Yellowish fluid	-224
	78.89	Male	25.50	Prosthetic knee	104.00	147.00	No	No	Delayed	No	Yes	Yes	Yes	Yellowish fluid	-224
11	83.02	Female	30	Prosthetic hip	78	87	No	No	Late	Yes	No	Yes	Yes	Purulence	-25
12	64.29	Female	50	Bilateral prosthetic knees	Unknown	Unknown	Yes	No	Late	Yes	Yes	Yes	Yes	Purulence	-4
	64.29	Female	50.00	Bilateral prosthetic knees	Unknown	Unknown	Yes	No	Late	Yes	Yes	Yes	Yes	None	-4
13	63.64	Male	33	Prosthetic hip	52	257	Yes	Toe amputation for osteomyelitis	Late	Yes	Yes	Yes	Yes	Purulence	3
14	72.72	Female	25	Prosthetic knee	Unknown	Unknown	Yes	No	Late	Yes	No	Yes	Yes	None	0
15	55.26	Male	28	Prosthetic Hip	114	87.60	No	No	Late	No	Yes	Yes	Yes	Purulence, sinus tract	-956
16	64.81	Male	28	Prosthetic hip	Unknown	Unknown	No	No	Late	Yes	No	Yes	No	Purulence, fluid collection	1092

Infectious and mortality outcomes

In our cohort of 16 patients, we identified seven cardiac device infections, eight native cardiac valve infections, and seven prosthetic/bioprosthetic valve infections, with some patients having multiple classifications of endocarditis simultaneously. In terms of microbiology, 10/18 (55.55%) of joints had methicillin-sensitive *Staphylococcus aureus* (MSSA) infection, and 3/18 (16.67%) of joints had infections due to MDROs, all of which were due to methicillin-resistant *Staphylococcus aureus* (MRSA). In addition, we found that 11/18 (61.11%) of joints in our cohort required oral antibiotic suppression therapy >6 months for indications that were determined through shared decision-making between the patient, infectious disease service, and orthopedic service. The rationale for prescribing suppressive antibiotics is documented in Table 4.

We also found that 13/16 (81.25%) of patients required cardiac surgery, but only one patient required repeat cardiac surgery in our study period, which was indicated for worsening heart failure in the setting of prosthetic valve dysfunction. The three patients who did not have cardiac surgery during the study period were determined to not meet the criteria for surgery according to mutual decisions made by the cardiothoracic surgery and infectious disease departments and were only treated with intravenous antibiotics. Lastly, 6/16 (37.5%) of patients died within two years of the endocarditis diagnosis, while 9/16 (56.25%) of patients died during the study period.

**Table 4:** Infectious Variables and Outcomes of Patients With Concurrent Endocarditis and PJI *Negative value means PJI was diagnosed prior to endocarditis. **Negative value means cardiac surgery was performed prior to PJI diagnosis. PJI: periprosthetic joint infection; DAIR: debridement, antibiotics, and implant retention; GBS: group B *Streptococcus*; MSSA: methicillin-sensitive *Staphylococcus aureus*; MRSA: methicillin-resistant *Staphylococcus aureus*; PICC: peripherally inserted central venous catheter; ICD: implantable cardioverter-defibrillator; PFO: patent foramen ovale; CRT-D: cardiac resynchronization therapy device

Patient number	Description of endocarditis	Days from endocarditis to PJI*	Infectious organism	Infectious organism MDR	Antibiotics used, duration, and route	Chronic oral antibiotic suppression after initial antibiotic treatment	Rationale for chronic oral antibiotic suppression, if used	Cardiac surgery	Cardiac surgery details, if applicable	Time from PJI diagnosis to cardiac surgery (days)**	Repeat cardiac surgery	Cardiac Surgery prior to PJI surgery
1	Pacemaker lead infection	15	Pseudomonas	No	Cefepime and levofloxacin for two weeks, through central line	No	n/a	Yes	Removal of left pectoral pacemaker with lead extraction, placement of a temporary pacing wire via the right internal jugular vein	2	No	No
2	Native valve endocarditis	-5	GBS	No	Penicillin for one week, though PICC line	No	n/a	No	Patient was not a surgical candidate for endocarditis given a risk of intracranial hemorrhage with anticoagulation	n/a	n/a	n/a
3	Pacemaker lead endocarditis, native aortic valve, native pulmonic valve endocarditis	324	MSSA	No	Oxacillin for six weeks, through PICC line	Yes	Six-month course of oral trimethoprim-sulfamethoxazole prior to reimplantation of hip prosthesis	Yes	Removal of infected pacemaker	-317	No	Yes
4	Pacemaker lead endocarditis, mobile descending aortic plaque infection	6	MSSA	No	Cefazolin for six weeks, though PICC line	Yes	Patient declined surgical management of PJI and was placed on indefinite oral cefadroxil	Yes	Pacemaker removal, reimplantation of new pacemaker at a later date	-6	No	Yes
5	Bioprosthetic aortic valve endocarditis	-5	Staphylococcus epidermidis	No	Six-week course of vancomycin and rifampin with two weeks of gentamicin, though PICC line	Yes	Patient only underwent DAIR and declined further surgical management; thus, chronic suppression was provided as an option with oral doxycycline daily	Yes	Redo sternotomy, redo aortic valve replacement, tricuspid valve repair	96	Yes, due to worsening heart failure	No
6	Mitral valve endocarditis	55	MSSA	No	Vancomycin for six weeks, ciprofloxacin for one week, gentamicin for one week, all through PICC line	Yes	Continued ciprofloxacin suppression for spontaneous bacterial peritonitis prophylaxis, continued intravenous vancomycin for months for arteriovenous fistula-related infection as well	No	Patient was not a candidate for cardiac surgery	n/a	n/a	n/a
7	ICD endocarditis, tricuspid valves endocarditis, PFO endocarditis	6	MSSA	no	IV oxacillin through PICC and oral rifampin for six weeks	Yes	Patient was placed on one year of suppressive oral cefuroxime after undergoing DAIR, as patient continued to have echocardiographic evidence of endocarditis; patient then underwent explant of his knee prosthesis but died prior to reimplantation	Yes	Extraction of ICD leads	6	No	Yes
8	ICD/pacemaker lead endocarditis	-3	MSSA	No	Cefazolin and rifampin for six weeks, though PICC line	Yes	Patient underwent DAIR but was not a candidate for two-stage revision; patient also continued to have chronic pectoral hematoma infection and thus was placed on oral minocycline and rifampin indefinitely	Yes	Removal of pacemaker and placement of semipermanent lead	3	No	No
9	Bioprosthetic mitral valve endocarditis	-143	MSSA	No	Nafcillin and rifampin for six weeks, through PICC	No	n/a	Yes	Midline sternotomy for redo mitral valve excision, debridement and replacement of mitral valve with a porcine bioprosthesis	176	No	No
10	CRT-D lead endocarditis, tricuspid and bioprosthetic aortic valve endocarditis	-224	MSSA	No	Nafcillin for six weeks, through PICC	Yes	Patient underwent DAIR of both knees but was not a candidate for two-stage revision; given this and a history of endocarditis, patient remained on oral doxycycline indefinitely	Yes	Revision sternotomy, aortic root replacement, tricuspid valve leaflet repair, ventricular septal defect closure, CRT-D explant	240	No	No
	CRT-D lead endocarditis, tricuspid and bioprosthetic aortic valve endocarditis	-224	MSSA	No	Nafcillin for six weeks, through PICC	Yes	Patient underwent DAIR of both knees but was not a candidate for two-stage revision; given this and a history of endocarditis, patient remained on oral doxycycline indefinitely	Yes	Revision sternotomy, aortic root replacement, tricuspid valve leaflet repair, ventricular septal defect closure, CRT-D explant	240	No	No
11	Bioprosthetic mitral valve endocarditis	-25	Staphylococcus mutans	No	Penicillin for six weeks, through PICC	No	n/a	No	Patient was not a candidate for cardiac surgery	n/a	n/a	n/a
12	Bioprosthetic aortic valve endocarditis	-4	MRSA	Yes	Vancomycin for six weeks, though PICC	Yes	Although the patient underwent two-stage revision with mega-endoprosthesis, she was placed on lifetime oral doxycycline given her MRSA endocarditis and comorbidities	Yes	Aortic root replacement, redo sternotomy	76	No	No
	Bioprosthetic aortic valve endocarditis	-4	MRSA	Yes	Vancomycin for six weeks, though PICC	Yes	Patient was placed on lifetime oral doxycycline given her MRSA endocarditis and comorbidities	Yes	Aortic root replacement, redo sternotomy	76	No	No
13	Native mitral and prosthetic aortic valve endocarditis and aortic root abscess	3	MSSA	No	Nafcillin, ceftriaxone, and rifampin for six weeks, through PICC	No	n/a	Yes	Reoperative aortic valve replacement, removal of vegetations from the mitral valve	-3	No	Yes
14	Native mitral valve endocarditis	0	MSSA	No	Nafcillin for six weeks, through PICC	No	n/a	Yes	Mitral valve repair	171	No	No
15	Tricuspid valve endocarditis and ICD endocarditis	-956	MRSA	Yes	Daptomycin and cefepime for six weeks, through PICC	Yes	Patient underwent DAIR and Girdlestone arthroplasty (due to proximal femoral fracture) and has remained on chronic oral minocycline due to endocarditis history, comorbidities, and continued hip drainage	Yes	ICD extraction	960	No	Yes
16	Bioprosthetic aortic valve endocarditis with a peri-annular abscess	1092	Enterococcus faecalis	No	Six-week course of intravenous ampicillin and gentamycin and oral linezolid	No	n/a	Yes	Aortic valve replacement, debridement and patching of periannular abscess	-1089	No	Yes

Orthopedic outcomes

**Figure 1:**
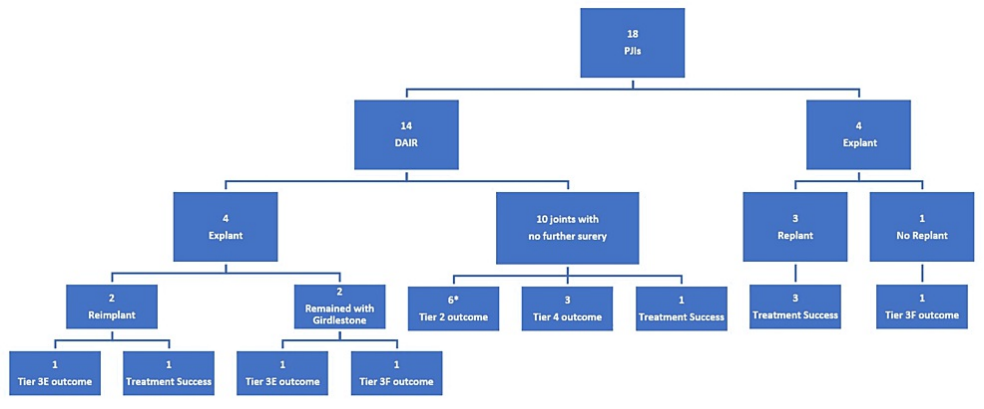
Treatment Outcomes for the 18 PJIs in Our Series Treatment outcome tiers were defined by Fillingham et al. [18]. Of note, one of the patients in this group, denoted by the “*” only had 12 months of clinical follow-up; all other patients had at least two years of clinical follow-up. PJI: periprosthetic joint infection; DAIR: debridement, antibiotics, and implant retention

In our cohort, we analyzed the outcomes of the 18 hip and knee PJIs that were diagnosed, as shown in Table 5. All PJIs were managed surgically, with 14/18 (77.77%) of joint infections initially being managed by DAIR and 4/18 (22.22%) of joint infections initially being managed by explant. Of the 14 joint infections managed with DAIR, 4/14 (28.57%) of joints subsequently required an explant, and 2/4 of these joints went on to reimplantation. Of the joints initially managed with explant, 3/4 (75%) went on to reimplantation. The three joints in our cohort that underwent explant of their prosthesis but did not complete the full two-stage revision were deemed either medically unfit for the reimplantation procedure or the patients did not have the desire to undergo further surgery. Only 5/18 (27.77%) of joints had a successful PJI treatment, while the remaining patients had varying tiers of failure. Notably, 7/18 (38.89%) of joints had treatment failure classified at or above tier 3E, which is representative of severe morbidity and mortality. The ultimate orthopedic outcome for each patient is documented in Table 5 and Figure 1.

**Table 5:** Orthopedic Outcomes of the 18 Joints Infected in Our Series **Negative value denotes that a PJI was diagnosed prior to endocarditis. Treatment failure is as defined by tier 2-4 outcome by Fillingham et al. [18]. DAIR: debridement, antibiotics, and implant retention; PJI: periprosthetic joint infection; PICC: peripherally inserted central venous catheter

Patient number	Type of prosthetic joint infected	Days from infected cardiac/vascular device to PJI**	Time from PJI diagnosis to first surgical management of PJI (days)	DAIR	Explant	Full two-stage revision, including reimplant	Ultimate outcome of infection	Treatment failure	Death within two years of endocarditis diagnosis	Category of last follow-up	Follow-up duration (months)
1	Prosthetic knee	15	0	Yes	No	No	Patient died during the hospitalization due to bacteremia	Yes, tier 4	Yes	Date of death	0.36
2	Prosthetic knee	-5	0	Yes	No	No	Patient died during hospitalization	Yes, tier 4	Yes	Date of death	0.76
3	Prosthetic hip	324	3	No	Yes	Yes	Patient successfully underwent two-stage revision of hip prosthesis	No	Yes	Date of death	4.10
4	Prosthetic hip	6	2	Yes	No	No	Patient declined further surgical intervention and remained on suppressive antibiotics until the date of death	Yes, tier 2	Yes	Date of death	4.80
5	Prosthetic knee	-5	0	Yes	No	No	Patient remained on suppressive antibiotics with PO doxycycline daily	Yes, tier 2	n/a	Clinical follow-up	12
6	Prosthetic knee	55	2	Yes	No	No	Patient died from bacteremia one year later while on multiple antibiotics for suppression therapy	Yes, tier 4	Yes	Date of death	13.66
7	Prosthetic knee	6	8	Yes	Yes	No	Patient died with antibiotic spacer in place in the right knee	Yes, tier 3F	Yes	Date of death	24
8	Prosthetic knee	-3	1	Yes	No	No	Patient remained on chronic suppressive antibiotics after DAIR	Yes, tier 2	No	Clinical follow-up	25.76
9	Prosthetic knee	-143	141	Yes	Yes	Yes	Patient underwent a Girdlestone arthroplasty followed by reimplantation of a knee prosthesis	No	No	Clinical follow-up	26.96
10	Prosthetic knee	-224	2	Yes	No	No	Patient remained on suppressive antibiotics for life	Yes, tier 2	No	Clinical follow-up	31.4
	Prosthetic knee	-224	2	Yes	No	No	Patient remained on suppressive antibiotics for life	Yes, tier 2	No	Clinical follow-up	31.4
11	Prosthetic hip	-25	1	No	Yes	Yes	Patient underwent a full two-stage revision	No	No	Clinical follow-up	46.56
12	Prosthetic knee	-4	2	Yes	Yes	Yes	Patient eventually had a radical resection left distal femur with removal of existing cement spacer followed by a distal femoral mega-endoprosthetic rotating hinge knee arthroplasty	Yes, tier 3E	No	Clinical follow-up	54.86
	Prosthetic knee	-4	2	Yes	No	No	Patient’s infection resolved after DAIR, patient remained on chronic suppressive antibiotics	Yes, tier 2	No	Clinical follow-up	54.86
13	Prosthetic hip	3	1	No	Yes	No	Patient underwent explant of hip components and spacer placement but did not have reimplantation	Yes, tier 3F	No	Date of death	64.43
14	Prosthetic knee	0	0	Yes	No	No	Patient’s infection resolved with six weeks of nafcillin via PICC line	No	No	Date of death	108.23
15	Prosthetic hip	-956	2	Yes	Yes	No	Patient underwent Girdlestone arthroplasty	Yes, tier 3E	No	Clinical follow-up	132.80
16	Prosthetic hip	1092	0	No	Yes	Yes	Patient underwent a full two-stage revision	No	No	Date of death	143.20

Survivorship analyses

We sought to determine if there was a significant difference in mortality within two years of PJI diagnosis and joint survivorship (defined as requiring explant of the prosthesis) between patients who had cardiac surgery performed prior to surgery for PJI management and patients who had surgery for PJI management prior to cardiac surgery. In both of these survivorship analyses, we were able to include a maximum of 13 patients, as three patients did not undergo cardiac surgery and were instead treated with intravenous antibiotics. In our analysis of mortality within two years of PJI, the two patients with synchronous PJIs were counted as one patient. We chose to do this because these two patients had the DAIRs for their synchronous PJIs performed on the same day. In our analysis of joint survivorship (requiring explant), we excluded patients who had explant as the original surgical management for their PJI (thus only including patients with DAIR as the original management).

**Figure 2:**
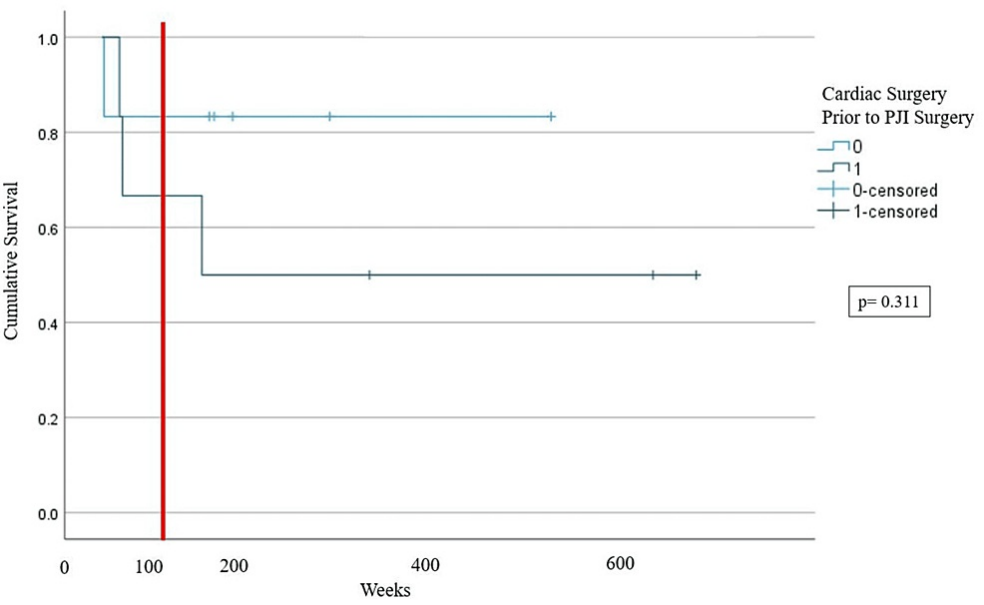
Kaplan-Meier Curve Depicting Mortality Within Two Years of PJI Two-year mortality is a function of whether cardiac surgery is performed prior to PJI surgery or vice versa. The large red vertical line depicts two years (104 weeks). 0: cardiac surgery was not before PJI surgery, 1: cardiac surgery was before PJI surgery PJI: periprosthetic joint infection

**Figure 3:**
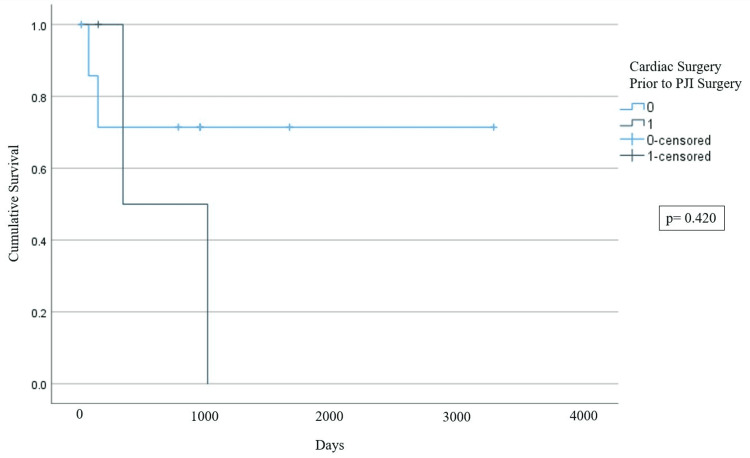
Kaplan-Meier Curve Depicting Joint Survival: Requiring Explant Requiring explant is a function of whether cardiac surgery is performed prior to PJI debridement, antibiotics, and implant retention (DAIR) or vice versa. 0: cardiac surgery was not before PJI surgery, 1: cardiac surgery was before PJI surgery PJI: periprosthetic joint infection

We found that mortality within two years appeared to be higher in patients who had surgery for PJI management prior to cardiac surgery, although the difference was not significant (log-rank p=0.311), as we were underpowered to detect a difference (Figure 2). We also found that joint survivorship appeared to be higher in patients who had PJI management surgery prior to cardiac surgery, but the results were not significant (log-rank p=0.420), again due to underpowering (Figure 3).

## Discussion

This study highlights the high morbidity and mortality in patients with concurrent PJI and infectious endocarditis. These concurrent diagnoses, while rare, do occur and should be ruled out in patients with bacteremia and symptoms of a PJI. Rakow et al. studied 106 hematogenous PJIs in a two-year period and found that, while the primary source of infection was identified in only 68% of cases, of the cases with an identifiable source, 19.44% of cases were due to infectious endocarditis [4]. This study shed light on the potential incidence of concurrent PJI and endocarditis cases but did not discuss any of the orthopedic outcomes of these patients. Furthermore, in a study by Tande et al. of 166 patients with at least one arthroplasty in place who were diagnosed with *Staphylococcus bacteremia*, 36.1% of patients developed at least one PJI, and of these PJI patients, three developed endocarditis [5]. In that study, only one of the three patients had recurrent PJI, but the overall outcome was not discussed. Therefore, a detailed review of our cohort’s infectious courses, treatment, and outcomes provides some insight into the seriousness of these concurrent diagnoses.

In our cohort, 87.5% of patients were diagnosed with a PJI classified as “late” (>24 months from index), which is consistent with the previous reporting of the incidence of patients who develop hematogenous PJI associated with another identified distant infection [12]. Within the first six months of PJI diagnosis, 25% (4/16) died of complications related to their infection, and one additional patient died of bacteremia just over a year after the initial PJI diagnosis. This patient who died 13 months after the PJI diagnosis was a male with untreated HIV and was on suppressive antibiotics. Of note, two out of the three patients who did not have cardiac surgery died from overwhelming infection, which is not surprising, as the primary focus of the infection (endocarditis) was not able to be fully addressed surgically. Overall, the mortality rate we found in our study is markedly higher than the reported one-year mortality rate after PJI alone of 3%-8% [7,9].

All 16 patients in our cohort underwent surgical intervention for the 18 PJIs. For the treatment of acute PJI with DAIR, the literature reports success rates of 83%-90% [19-21]. However, in our cohort, 2/14 (14.28%) patients undergoing DAIR had treatment success, and only 5/18 (27.77%) of all of the joints had successful PJI management. We found that, in total, 8/18 joints required explant at any point in time, and of these eight joints, three never went on to reimplant, at a rate of 37.5%. This rate of failure to undergo reimplant is higher than the reported attrition rates after the first stage of a two-stage revision, which are normally around 18% [22]. Determining the optimal surgical treatment should continue to be case-specific depending on factors including the chronicity of infection, presence of systemic infection, patients’ medical comorbidities, and surgeon’s preference. Nevertheless, patients should be counseled on the increased risks of treatment failure with either DAIR or two-stage revision, as 72.22% of all of the joints in our cohort had a failed treatment for PJI.

Regarding survivorship of patients as a function of whether cardiac surgery or PJI management surgery (DAIR of explant) was performed first, we were unable to identify significant differences. While this is due to insufficient power, it is interesting to note that in the three joints that had a cardiac surgery prior to DAIR, two required explant, and all had treatment failure. Previous research has shown that a shorter interval time between infectious symptoms and DAIR of the affected prosthetic joint is associated with improved joint survivorship (due to decreased biofilm formation) [23,24]. While performing cardiac surgery for endocarditis might delay DAIR for PJI, thus increasing the risk for treatment failure, addressing immediate life-threatening conditions such as hemodynamic instability and acute heart failure likely takes precedent to debriding the joint. Given that treatment failure was also high in patients who had PJI surgery prior to cardiac surgery, we suspect that the high treatment failure in our cohort is representative of the overall gravity of these patients’ medical conditions, regardless of whether one surgery took precedent during the hospitalization.

Despite only including cases in which the same organism was identified in both cardiac infection and PJI, for most patients in the cohort, we could not identify an underlying impetus for their infection. Two patients were noted to have had recent foot-related procedures that may have caused a hematogenous infection, and one had a recent pacemaker exchange that became infected. Notably, no patients had any documented recent dental procedures, for which antibiotic prophylaxis is recommended by many orthopedic and cardiac surgeons for both infectious endocarditis and prosthetic joint infection prevention in the setting of preexisting prosthetic implants [25,26]. Additionally, no patients had a documented history of injection drug use despite the high association with infectious endocarditis [27].

The most commonly identified organism in our cohort was *Staphylococcus aureus*, with MRSA in 16.67% of cases and MSSA in 55.55% of cases, for a total of 72.22% of organisms identified. This is consistent with previous literature describing that *Staphylococcus aureus* has some of the highest rates of seeding a periprosthetic joint in the setting of bacteremia compared to other organisms, at 18%-21% likelihood of seeding [28,29]. Nevertheless, in the literature, MSSA PJI has high rates of two-year infection-free survival, reported at 93% in one study [30]. In our cohort, 33% (3/9) of patients with MSSA infection died related to the infection, highlighting the high mortality risk with these concurrent infections of PJI and endocarditis.

Limitations

Although the sample size of this study represents the largest known cohort of this rare concurrent pathology, the limitations of this study include the retrospective design and the small size of our cohort. A larger cohort could potentially allow us to compare and make conclusions regarding outcomes between the initial explant versus DAIR. We lacked sufficient cohort size to identify a statistically significant difference in survivorship between those who had their PJI surgical treatment before versus after the intervention of their cardiac infection. As a chart review-based study, we were limited to only information documented in their medical record; therefore, questions that may have suggested a possible source of infection such as dental procedure or injection drug use may not have been asked or documented.

Currently, there is no consensus diagnostic process for determining whether endocarditis or PJI was the initial infection in the setting of very short intervals between the diagnoses, which limits the interpretability of our study to the outcomes of patients who have “concurrent infections.” Furthermore, while we included only patients who had a shared identified organism from both the cardiac infection and PJI to identify a relationship between the infections, we may have created a selection bias against difficult-to-culture organisms, which may explain the predominance of MSSA infections that were identified.

## Conclusions

Concurrent infectious endocarditis and prosthetic joint infection is associated with high morbidity and mortality. Of the 18 PJIs, 72.23% resulted in treatment failure, defined as any outcome equal to or worse than requiring chronic suppressive antibiotics. Of the joints that underwent explant, three never went on to reimplant, at a rate of 37.5%. Patients with these two concurrent infections should be counseled that not only the associated mortality rate is high, but also the surgical treatment of their PJI has a high rate of treatment failure, including an explant following an initial DAIR, an explant with retained spacer, or a requirement of lifelong antibiotic suppression.
